# Transcatheter Closure of a Paravalvular Leak Guided by Transesophageal Echocardiography and Three-Dimensional Printing

**DOI:** 10.3389/fcvm.2022.750896

**Published:** 2022-05-20

**Authors:** Chennian Xu, Yang Liu, Mengen Zhai, Ping Jin, Lanlan Li, Yanyan Ma, Jian Yang

**Affiliations:** Department of Cardiovascular Surgery, Xijing Hospital, Air Force Medical University, Xi’an, China

**Keywords:** paravalvular leak, transesophageal echocardiography, three-dimensional printing, transcatheter closure, follow-up

## Abstract

**Background:**

Closure of a percutaneous paravalvular leak (PVL) is a technically challenging procedure because of the specific anatomy postoperatively and the complex catheter techniques required. Transesophageal echocardiography (TEE) and three-dimensional (3D) printing might be helpful in identifying complex anatomical structures and the procedural design.

**Objectives:**

The purpose of this study was to review our experiences with transcatheter closure of PVL guided by TEE and 3D (TEE&3D) printing.

**Methods:**

A total of 166 patients with PVL after surgical valve replacement underwent transcatheter closure, from January 2015 through December 2020. Among these patients, 68 had preoperative guidance from TEE&3D printing. We reviewed the catheter techniques, perioperative characteristics, and prognosis. The median follow-up period was 36 (3–70) months.

**Results:**

Acute procedural success was achieved in 154/166 (92.8%) patients; of these, 64/68 (94.1%) had TEE&3D guidance and 90/98 (91.8%) had transthoracic echocardiography (TTE) guidance. No hospital deaths occurred. All patients having percutaneous procedures were given local anesthesia, while 13 patients having transapical procedures were given general anesthesia. Multiple approaches were used, including transfemoral, transapical, and transseptal *via* the arteriovenous loop. We also deployed multiple devices, including the Amplatzer Vascular Plug II (AVP II), the Amplatzer duct occluder II, the patent ductus arteriosus (PDA) occluder, and the Amplatzer muscular ventricular septal defect occluder. Those cases guided by TEE&3D printing had shorter procedural times compared with those guided by TTE [(61.2 ± 23.4) vs. (105.7 ± 53.9) min, *p* < 0.05]. The fluoroscopic time was also shorter for operations guided by TEE&3D printing compared with those guided by TTE alone [(18.5 ± 11.4) vs. (27.3 ± 5.6) min, *p* < 0.05]. The complications included recurrent hemolysis, residual regurgitation, acute renal insufficiency, and anemia. There was no significant difference in the incidence of complications between the 2 groups.

**Conclusion:**

Transesophageal echocardiography and 3D printing show advantages compared with standalone TTE in guiding the transcatheter closure of PVL with shorter procedural and fluoroscopic times. This minimally invasive treatment could provide reliable outcomes in selected patients.

**Clinical Trial Registration:**

[www.ClinicalTrials.gov], identifier [NCT02917980].

## Introduction

Paravalvular leakage (PVL) is a unique complication after heart valve replacement and the most common cause of reoperation after valve replacement, with an incidence of 0.75–2.3% (1). The proportion of cases with perivalvular leakage of the mitral valve was significantly higher than that of cases with perivalvular leakage of the aortic valve (2). Moderate and severe PVL can cause serious adverse events such as progressive cardiac insufficiency, hemolytic anemia, and infective endocarditis. In severe cases, the prosthetic valve may tilt, swing, dissociate, or even fall off, requiring emergency surgery (3). Among patients with PVL, approximately 3% require treatment because of congestive heart failure or hemolytic anemia (4–7). Surgery with repair or re-replacement was the classical treatment for PVL. Recently, transcatheter closure of PVL has emerged as an alternative treatment for patients with a high surgical risk (8–10). In our experience, three-dimensional (3D) printing for preoperative evaluation can improve the success rate. This study compared transthoracic echocardiography (TTE) and transesophageal echocardiography (TEE) and 3D (TEE&3D) printing in the diagnosis and differential diagnosis of PVL after heart valve replacement to explore the value of TEE&3D printing in the diagnosis and preoperative evaluation of PVL, improve the success rate of interventional therapy for PVL, reduce complications, and provide an important basis for postoperative follow-up. This retrospective study presents the perioperative outcomes and midterm follow-up results of the transcatheter closure of PVL.

## Materials and Methods

### Study Design and Patient Population

**FIGURE 1:**
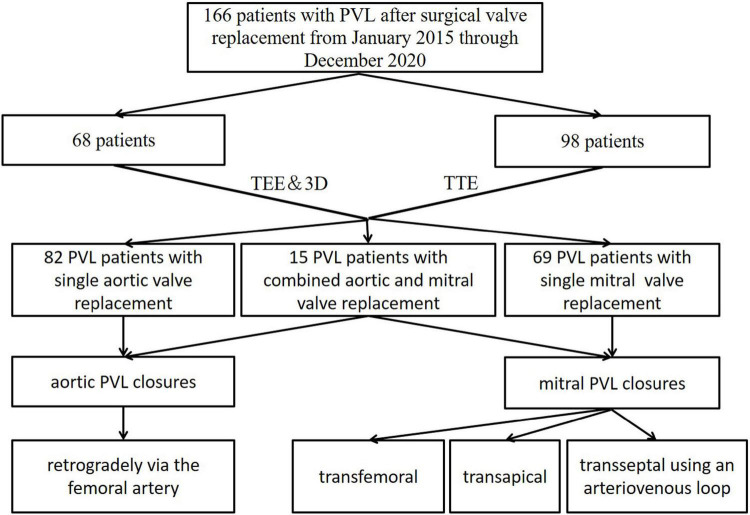
Patient flow diagram of the transthoracic echocardiographic (TTE) group and the transesophageal and 3-dimensional group.

The protocol of the study was approved by the Ethics Committee of Xijing Hospital, Air Force Medical University (KY20150205-1) (Figure 1).

A total of 166 patients with PVL after surgical valve replacement underwent transcatheter closure at Xijing Hospital in China, from January 2015 to December 2020. All 166 patients or guardians of patients provided informed consent to participate in the study, and all clinical documents were reviewed for analysis.

A total of 151 patients had single prosthetic valve replacements, and 15 patients had previously combined aortic and mitral prosthetic valve replacements. A total of 136 patients had mechanical valves, and 30 patients had bioprosthetic valves. The patients were advised of the procedural risks and options as well as of the off-label use of all closure devices. Patient demographics and medical histories are shown in Table 1.

**TABLE 1:** Preoperative clinical characteristics.

Variables	Patients (*n* = 166)
Gender, male	119 (71.7%)
Age, years	57.3 ± 8.31
**Previous procedure**	
Aortic valve replacement	62 (37.3%)
Mitral valve replacement	47 (28.3%)
Combined aortic and mitral valve replacement	57 (34.3%)
Time since valve replacement, years	4.25 ± 2.92
History of endocarditis	41 (24.7%)
Hemolysis	101 (61.2%)
NYHA FC II	57 (34.3%)
NYHA FC III	89 (53.6%)
NYHA FC IV	20 (12.0%)
**LVEF**	
< 40	29 (17.4%)
40–50	74 (44.6%)
> 50	63 (38.0%)
**PVL severity**	
Mild	5 (3.0%)
Moderate	45 (27.1%)
Moderate to severe	82 (49.4%)
Severe	34 (20.5%)
**Comorbidities**	
Atrial fibrillation	44 (38.9%)
Coronary artery disease	9 (8.0%)
Pulmonary hypertension	26 (23.0%)
Systemic hypertension	22 (19.5%)
Chronic renal insufficiency, Creatinine > 1.5 mg/dL	12 (10.6%)
**EuroSCORE II**	
0–2	10 (6.0%)
3–5	101 (60.8%)
> 6	55 (33.2%)

*Categorical variables are presented as frequency (%); continuous variables are presented as mean ± standard deviation when normally distributed. The degree of paravalvular regurgitation was graded semi-quantitatively using Doppler echocardiography and color-flow imaging (mild: < 5 ml; moderate: 5–8 ml; moderate to severe: 8–12 ml; severe: > 12 ml). When multiple jets were present, the amounts of regurgitation from the separate jets were totaled for semi- quantitation. NYHA FC, New York Heart Association functional class; LVEF, left ventricular ejection fraction; PVL, paravalvular leak.*

### Transthoracic Echocardiography and Transesophageal Echocardiography

**FIGURE 2:**
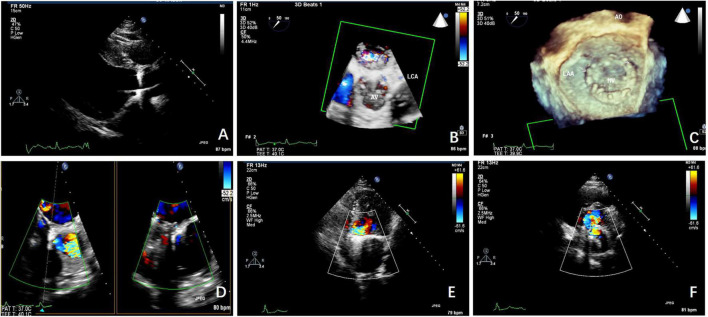
The TTE and transesophageal echocardiographic (TEE) scans taken before the procedure. **(A)** TEE shows the aortic paravalvular leakage (PVL) before the procedure. **(B)** 3-Dimensional (3D)-TEE shows the aortic PVL before the procedure. **(C)** 3D-TEE shows the mitral PVL before the procedure. **(D)** The mitral PVL as seen on the TEE scan. **(E)** The mitral PVL as seen on the TTE scan. **(F)** The mitral PVL was as seen on TTE.

All transcatheter procedures were performed in the catheterization laboratory. The location of the PVL and the volume of regurgitation were confirmed by TEE and 3D printing technology before the procedures in the TEE&3D group. Thirteen mitral PVL closures were performed *via* the transapical approach with the patients under general anesthesia. All the other 151 procedures were performed with the patients under local anesthesia. Multiple approaches were used, including transfemoral, transapical, and transseptal, *via* an arteriovenous loop, according to the anatomy, the location of the PVL, and previous operation(s) (Figure 2).

### Three-Dimensional Printing

The original CT angiography imaging data of the patients were imported into Materialise Mimics 21.0 software (Materialise Company, Belgium), and the end systolic image was selected. The threshold was to select the region of interest and build a mask. The mask was constructed in combination with the functions of region growth, split mask, and edit masks, and the dimensions of the 3D model, including the valve and the surrounding anatomical structures, were calculated. The 3D model was exported in Standard Tessellation Language format and imported into 3-Matic (Materialise Company, Belgium) software to obtain Standard Tessellation Language format files that could be used for 3D printing. A translucent rubber material (Agilus 30 Clear) was used to print the 3D model of PVL using a Stratasys J750 printer (Stratasys, Eden Prairie, MN, United States).

**FIGURE 3:**
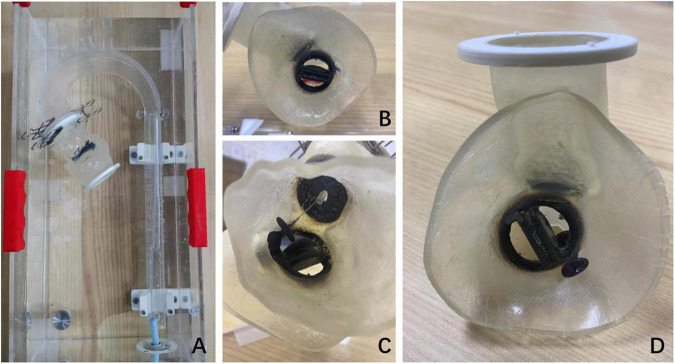
Preoperative 3D printing model and preoperative simulation *in vitro*. **(A)** The preoperative plan was simulated in an external phantom using the 3D printing model. **(B)** The mitral PVL and its position as seen in the 3D printing model. **(C)** The implantation of the occluder in the mitral PVL was performed retrogradely *via* the femoral artery in the 3D printing model. **(D)** The 3D model shows the situation after the occluder was implanted in the mitral PVL.

Three-dimensional printing models of the anatomical structure of the PVL were reconstructed according to the preoperative CT results in the TEE&3D group, which could assist the operator to observe more intuitively the location and shape of the PVL. The operation could be planned before the procedure with the help of 3D printing models in a simulator *in vitro* (Figure 3).

### Procedure

All transcatheter procedures were performed in the biplane catheterization laboratory. The location of the PVL and the volume of the regurgitation were confirmed by TEE combined with 3D printing models or TTE, before the procedures were carried out in selected patients. Thirteen mitral PVL closures were performed *via* the transapical approach with the patients under general anesthesia. All the other 153 procedures were performed with the patients under local anesthesia. All aortic PVL closures were approached retrogradely *via* the femoral artery. The mitral PVL closures were performed *via* multiple approaches, including transfemoral, transapical, and transseptal, using an arteriovenous loop.

### Transcatheter Closure of an Aortic Mechanical Paravalvular Leak

**FIGURE 4:**
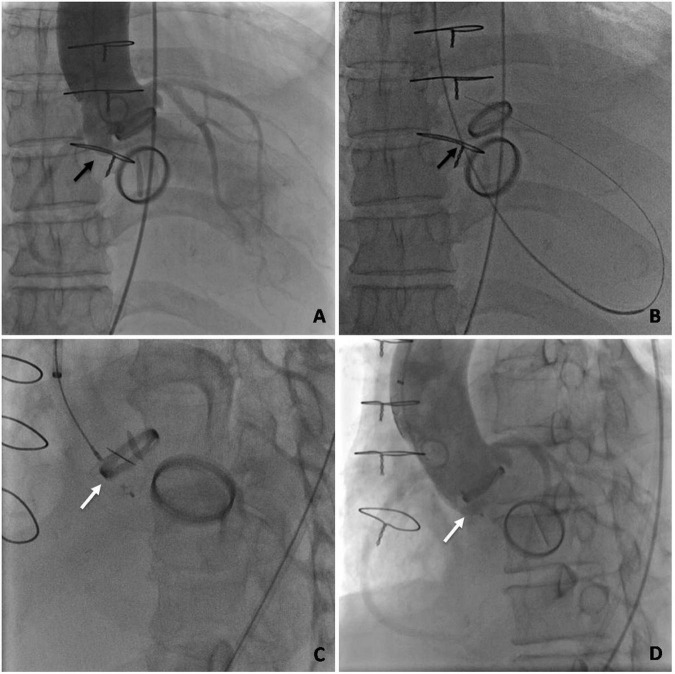
Angiography profiling of the transcatheter closure of an aortic mechanical PVL. **(A)** Ascending aorta angiogram to profile para-aortic regurgitation. **(B)** Retrograde crossing of the PVL with a guidewire. **(C)** An occluder placed at the position of the PVL. **(D)** Ascending aorta angiogram after deployment. (The black arrow indicates the PVL. The white arrow indicates the occluder).

The procedures were performed retrogradely *via* the femoral artery in all patients with an aortic PVL. Using 6 Fr arterial access, a 5 Fr multipurpose diagnostic catheter (Boston Scientific Corporation, Marlborough, MA, United States) and a 260-cm (0.032-in) straight-tip wire (Terumo Corporation, Tokyo, Japan) were advanced through the PVL after an initial aortic angiographic scan confirmed the aortic regurgitation and the location of the PVL. An extra stiff, 0.035-inch exchange-length Lunderquist guidewire (Cook Medical, Bjaeverskov, Denmark) was then placed through the defect into the left ventricle, followed by the placement of a relatively larger transducing sheath (e.g., 6, 7, or 8 Fr Cook sheath [Cook Medical]) over the guidewire, through which the appropriate Amplatzer occluder device (AGA Medical Corp., Plymouth, MN, United States) was deployed. Multiple devices may be deployed simultaneously depending on the size and shape of the defect (Figure 4).

### Transcatheter Closure of a Mitral Mechanical Paravalvular Leak

**FIGURE 5:**
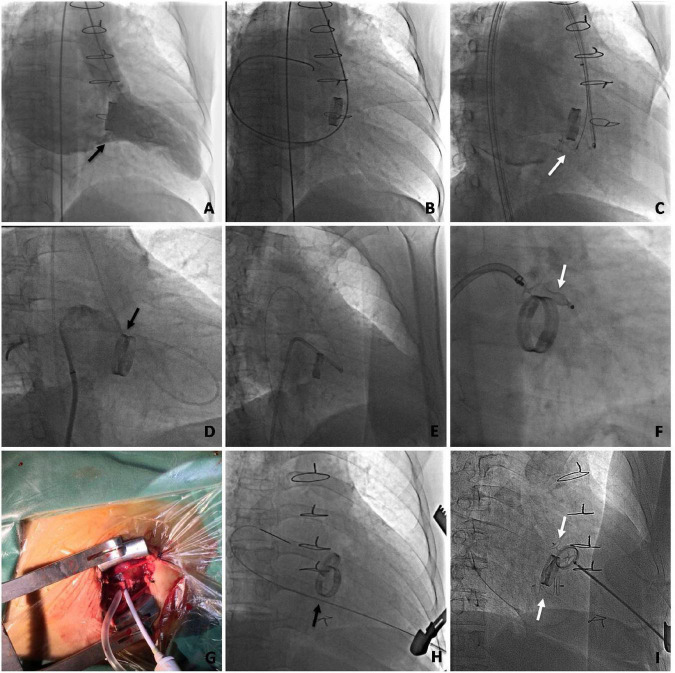
Angiography during the transcatheter procedure of mitral mechanical PVL closure *via* multiple approaches. **(A–C)** Transfemoral retrograde approach. **(A)** Left ventricular angiogram to profile paramitral regurgitation. **(B)** Retrograde crossing of the PVL with the guidewire. **(C)** Occluder placed at the position of the PVL. **(D–F)** Arteriovenous wire loop approach. **(D)** The retrograde crossing of the PVL with the guidewire followed by a transseptal puncture. **(E)** The sheath is advanced into the left ventricle from the femoral vein *via* the arteriovenous wire loop. **(F)** An occluder is placed at the position of the PVL. **(G–I)** Mini-invasive transapical approach. **(G)** The transapical access is obtained with a 6 Fr sheath. **(H)** The mitral PVL crossed retrogradely with the guidewire. **(I)** The occluder is deployed. (The black arrow indicates the PVL. The white arrow indicates the occluder).

The retrograde approach *via* the femoral artery was the first choice for patients with pure mitral valve replacement. The left ventricular angiogram confirmed the paramitral regurgitation and the location of the defect, after a 6 Fr pigtail catheter was placed in the left ventricle *via* femoral arterial access. A 5 Fr multipurpose diagnostic catheter and a 260-cm (0.032-in) Terumo straight-tip wire were then advanced through the defect based on the results of the angiogram. An extra stiff, 0.035-inch exchange-length Lunderquist guidewire was placed through the aortic valve and the paramitral defect into the left atrium, followed by the placement of a relatively larger transducing sheath over the guidewire. The appropriate Amplatzer occluder device was then deployed (Figure 5).

We used various devices for percutaneous closure of PVLs including the patent ductus arteriosus (PDA) occluder, the Amplatzer muscular ventricular septal defect occluder, the Amplatzer duct occluder II, and the Amplatzer Vascular Plug II (AVP II) (AGA Medical Corp., Plymouth, MN, United States).

### Clinical Follow-Up

All clinical files were reviewed, and perioperative characteristics were documented, including procedural time, fluoroscopic time, blood transfusions, perioperative laboratory blood tests, and postoperative hospital stay. All patients were seen in the clinic to ascertain their clinical status (New York Heart Association functional class) and adverse events after discharge. TTE was performed to evaluate the improvements in the construction and function of the patients’ hearts at 1, 3, 6, and 12 months after the procedure. CT angiography was also performed during the follow-up period.

### Statistical Analyses

All demographic, valve-related, procedural, and outcome data and clinical and anatomical data were obtained from a retrospective review of patient charts and procedural records. Statistical analyses were conducted using SPSS 22.0 software (IBM Corp., Armonk, NY, United States). Continuous variables are presented as means ± SD and categorical variables are expressed as percentages. Univariable comparisons were performed with the Student unpaired *t*-test for continuous normally distributed data and the χ^2^-test for categorical data. Values of *p* < 0.05 were considered statistically significant.

## Results

### Procedural and In-Hospital Outcomes

Acute procedural success was achieved in 154/166 (92.8%) patients, with 64/68 (94.1%) being patients with TEE&3D guidance and 90/98 (91.8%) being patients with TTE guidance (TEE&3D vs. TTE, *p* > 0.05). There were no hospital deaths. All patients who had percutaneous procedures were given local anesthesia, while the 13 patients who had transapical procedures received general anesthesia.

Transesophageal echocardiography and 3D-guided cases had shorter procedural times, with 61.2 ± 23.4 vs. 105.7 ± 53.9 min (*p* < 0.05) in TTE-guided cases. The fluoroscopic time was also shorter in TEE&3D-guided cases with 18.5 ± 11.4 vs. 27.3 ± 15.6 min (*p* < 0.05) in TTE-guided cases. The complications included recurrent hemolysis, residual regurgitation, acute renal insufficiency, and anemia. There was no significant difference in the incidence of complications between the 2 groups. Multiple devices were used to close the PVL, including PDA occluders, muscular ventricular septal defect occluders, and AVP II occluders. In 12 patients in the TEE&3D and TTE groups, an AVP II or an Amplatzer II duct occluder was deployed at the defect. However, in 1 case, the occluder could not be stabilized at the defect and could be easily pulled back into the aorta or the left atrium in a push-pull test, at which point the procedure was terminated and the patient underwent open surgery later. There were no hospital deaths. The procedural characteristics are shown in Table 2.

**TABLE 2:** Procedural characteristics of the transthoracic echocardiographic (TTE) group and the transesophageal echocardiographic (TEE) and 3-dimensional group.

	Total patients	TEE&3D	TTE	*P*-value (TEE&3D vs. TTE)
Acute successful procedures	154/166 (92.8%)	64/68 (94.1%)	90/98 (91.8%)	0.2638
Aortic PVL	93 (56.0%)	36 (52.9%)	57 (58.2%)	0.1343
Mitral PVL	72 (43.4%)	31 (45.6%)	41 (41.8%)	0.2637
Combined aortic and mitral PVL	1(0.6%)	1(1.5%)	0	
Approach of aortic PVL				0.1053
Transfemoral	94	37	57	
Approach of mitral PVL				0.6328
Transfemoral	30(41.2%)	12	18	
Trans-septal	25(34.2%)	11	14	
Transapical	13(17.8%)	6	7	
Transseptal A-V loop	5(6.8%)	3	2	
Devices				0.6207
AVP II occluder	96	36	60	
ADO II	38	13	25	
PDA occluder	5	1	4	
VSD occluder	15	6	9	
Number of devices				0.6033
Single device	121	48	73	
Two devices	29	14	15	
Three devices	4	2	2	
General anesthesia	13	6	7	0.2455
Local anesthesia	153	62	89	0.5807
Fluoroscope time (min)	27.4 ± 16.3	18.5 ± 11.4	27.3 ± 5.6	0.0238
Procedural time (min)	102.5 ± 49.6	61.2 ± 23.4	105.7 ± 53.9	0.0042
Hospital stay (days)	8.2 ± 3.3	7.3 ± 3.1	10.2 ± 4.7	0.6873
Patients needing blood transfusion	16 (10.3%)	5 (10.3%)	11 (10.3%)	0.2587

*A-V, arteriovenous; PDA, patent ductus arteriosus; ADO, Amplatzer duct occluder; VSD, ventricular septal defect; AVP, Amplatzer vascular plug.*

In this study, the volume of PVL regurgitation decreased to mild and moderate-mild immediately after the procedure in 154 patients of both the TEE&3D and the TTE groups, who were treated successfully. Eleven patients had hemolysis after the procedure. Of these, 7 patients had acute renal insufficiency and needed continuous renal replacement therapy and blood transfusions. All of these patients recovered before being discharged from the hospital. Other complications included 3 femoral pseudoaneurysms and 1 hemothorax after the transapical approach. All of these patients also recovered before discharge from the hospital.

### Follow-Up

**FIGURE 6:**
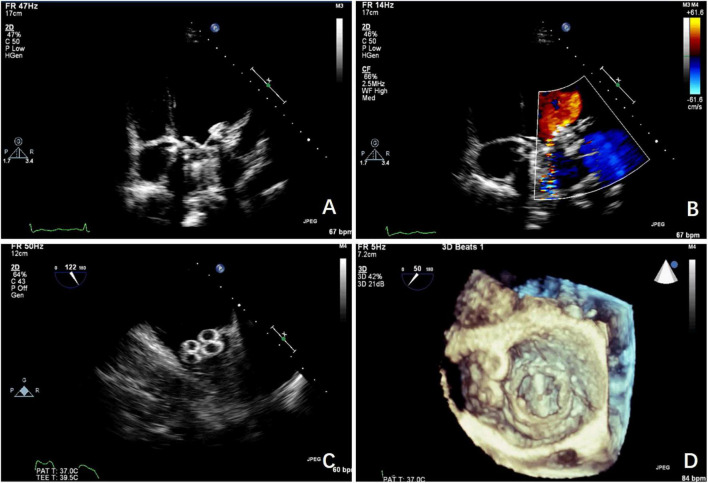
Echocardiograms taken during the follow-up period. **(A)** TTE shows the mitral PVL after the procedure. **(B)** TTE shows the mitral PVL closed with the occluder. **(C)** The mitral PVL is closed with 2 occluders with TEE guidance. **(D)** The mitral PVL is closed with 2 occluders under 3D TEE guidance.

Most patients no longer had mild to moderate paravalvular regurgitation during the follow-up examination with TEE, TTE, or CT angiography, 1–12 months after the procedure (Figure 6).

**FIGURE 7:**
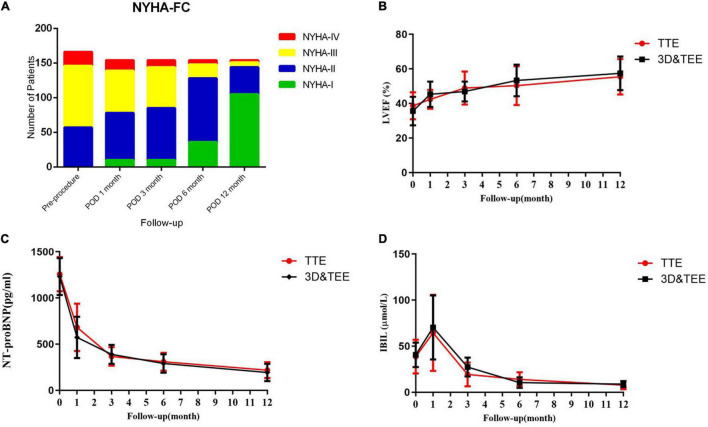
The 1-year follow-up of TTE and transesophaeal echocardiography and the 3-dimensional (TEE&3D) groups. **(A)** Improvement of the New York Heart Association functional class during the 1-year follow-up period. **(B)** The left ventricular ejection fraction during the 1-year follow-up period (TEE&3D vs. TTE, *p* > 0.05). **(C)** NT-proBNP levels during the 1-year follow-up period (TEE&3D vs. TTE, *p* > 0.05). **(D)** Indirect bilirubin levels during the 1-year follow-up period (TEE&3D vs. TTE, *p* > 0.05).

The median follow-up period was 36 (3–70) months, and follow-up was 100% complete. A total of 105 (68.2%) patients improved by ≥ 1 New York Heart Association functional class at the 1-year follow-up visit. The left ventricular ejection fractions showed no significant improvement in the TEE&3D vs. the TTE groups (*p* > 0.05). However, the levels of N-terminal pro-brain natriuretic peptide (NT-proBNP) returned to normal in most patients (TEE&3D vs. TTE, *p* > 0.05). The indirect bilirubin level decreased significantly after the procedure (TEE&3D vs. TTE, *p* > 0.05). Furthermore, there were no significant differences between the TEE&3D and the TTE groups in the foregoing follow-up (TEE&3D vs. TTE, *p* > 0.05) comparisons (Figure 7).

Four patients had recurrent hemoglobinuria in the first 2 months after discharge. Two of them had severe anemia. The valve was re-replaced for the 2 patients, 2 months after discharge. The occluder interfered with the disk of the mechanical valve. One patient died of low cardiac output syndrome after open-heart surgery. The other patients recovered uneventfully within 3 months.

## Discussion

Paravalvular leakage is a common complication after surgical valve replacement. Among patients with PVL, approximately 3% require treatment because of heart failure or hemolysis (11, 12). Since interventional catheter technology has been used in the treatment of perivalvular leakage, the treatment of perivalvular leakage has less traumatic choices (13, 14). The interventional occlusion of PVL is one of the most difficult operations in the treatment of structural heart disease. The operation often takes a long time, has high requirements for interventional techniques such as catheter guidewire manipulation, and needs the assistance of special types of equipment. Therefore, the technical success rates were limited to 67–90% according to previous reports in the literature (15, 16). The complicated pathological anatomy could be the main reason for the difficulties with the procedures. Also, surgeons working in heart centers with limited cases required longer learning curves. Currently, echocardiography and CT are used most often for monitoring the procedures. However, they might not be sufficient for creating the ideal procedural design and improving the technical success rate.

With the continuous progress of medical technology and imaging modalities, a medical model becomes more and more important. TEE&3D printing can generate a model that conforms to the characteristics of individual patients. It can help the doctors to plan the operation carefully without the patient. It is also a valuable teaching aid (17, 18). 3D printing technology and models will become an interpretation tool for doctors and patients to exchange professional knowledge. The preoperative 3D TEE examination and the creation of 3D printing models also contribute significantly to surgical risk assessment and the prevention of complications (19, 20). In addition, the harmonious doctor–patient communication created by using the 3D printing model not only provides information about treating diseases and improves the cure rate of diseases but also helps resolve misunderstandings and contradictions between doctors and patients and reduces the occurrence of medical accidents (21). In patients needing PVL closure, the preoperative 3D TEE examination and 3D printing model can help physicians to understand the anatomical details of the individual leak. They can then make a specific plan for each patient before the operation, including the choice of approach, the type and size of occluders, and a plan for preventing potential complications. 3D printing can also create a model for *in vitro* simulation, which could be used to verify the reliability and safety of the procedural plans.

In this study, TTE, TEE, and 3D printing were used for the preoperative diagnosis and to obtain information for image evaluation before carrying out the procedures. A total of 166 PVL patients were included in the study, which comprised relatively more cases for PVL interventional therapy (14, 15). The surgical plan was based on these findings. Five different approaches were used for aortic and mitral PVL closures. The first-line approach varied with each patient. All previous surgical details were collected and analyzed before performing the procedure, including which kind of prosthetic valve was implanted, whether it was a combined aortic valve replacement, and whether or not the atrial septum was sutured. The location, the size, and the structure of the PVL were confirmed by TEE, TTE, and the 3D printing models. The first-line approach was chosen on the basis of all these diagnostic details. We used the transfemoral approach if the patient had mitral valve replacement only. We used the retrograde transfemoral artery as the first-line approach, if the mitral PVL was located at around 6 o’clock, and the anterograde transseptal approach if the mitral PVL was located at around 12 o’clock. Therefore, to choose the appropriate approaches may make the delivery sheath easier to advance. Although there were no significant differences between the TEE&3D and the TTE groups in the follow-up of the left ventricular ejection fractions and the NT-proBNP and the indirect bilirubin levels, the TEE&3D-guided cases had shorter procedural times than the TTE-guided cases. The fluoroscopic time was also shorter in the TEE&3D-guided cases than in TTE-guided cases.

The other problem with interventional treatment of perivalvular leakage is that the types of special percutaneous delivery system and specialized equipment are relatively few (22–24). The Occlutech device and the Amplatzer vascular plug III are specific devices for PVL. At present, the devices used actually, such as the Amplatzer atrial septal defect and the ventricular septal defect occluders, the PDA occluder, and the AVP II, are designed for other heart diseases and are therefore not completely suitable for the treatment of perivalvular leakage. The TEE&3D printing techniques were able to provide more detailed anatomical data that could help the doctors and biomedical engineers understand the anatomy of the perivalvular leakage. This approach will facilitate the design and development of a new generation of dedicated devices for PVL closure and delivery systems.

### Limitations

The present study is a retrospective, non-randomized study from a single center with its inherent limitations. The physicians needed to go through a learning curve to become familiar with the technique of the transcatheter PVL closure. Furthermore, the follow-up time was limited and our experience with 3D printing is still preliminary. In any case, further studies are necessary to evaluate the long-term results.

## Conclusion

Transcatheter PVL closure requires complex catheter techniques. TEE and 3D printing help shorten the procedural and fluoroscopic times for transcatheter closure of PVL. This minimally invasive treatment could provide reliable outcomes in selected patients.

## Data Availability Statement

The raw data supporting the conclusions of this article will be made available by the authors, without undue reservation.

## Ethics Statement

The studies involving human participants were reviewed and approved by the Ethics Committee of Xijing hospital. The patients/participants provided their written informed consent to participate in this study.

## Author Contributions

CX acquired, analyzed the data, and wrote the manuscript. YL and MZ acquired, analyzed, interpreted the data, and revised the manuscript. JY designed the study, acquired, analyzed, and interpreted the data. PJ, LL, and YM revised the manuscript and acquired the data. All authors read and approved the final manuscript.

## Conflict of Interest

The authors declare that the research was conducted in the absence of any commercial or financial relationships that could be construed as a potential conflict of interest.

## Publisher’s Note

All claims expressed in this article are solely those of the authors and do not necessarily represent those of their affiliated organizations, or those of the publisher, the editors and the reviewers. Any product that may be evaluated in this article, or claim that may be made by its manufacturer, is not guaranteed or endorsed by the publisher.

## Abbreviations

3D: three-dimensional
ADOII: Amplatzer duct occluder II
AVPII: Amplatzer vascular plug II
PDA: patent ductus arteriosus
PVL: percutaneous paravalvular leak
TEE: transesophageal echocardiography
TTE: transthoracic echocardiography.
